# Induced Mammary Epithelial Cell-Derived Extracellular Vesicles Promote the Repair of Skin Trauma

**DOI:** 10.3390/ijms26209929

**Published:** 2025-10-12

**Authors:** Siyao Pan, Dandan Zhang, Guodong Wang, Longfei Sun, Mengzhen Wei, Shan Deng, Jianwei Chen, Prasanna Kallingappa, Xiang Yuan, Ben Huang

**Affiliations:** 1 Guangxi Key Laboratory of Eye Health, Guangxi Academy of Medical Sciences, Nanning 530021, China; 18434763568@163.com (S.P.);; 2 School of Life Science and Engineering, Southwest Jiaotong University, Chengdu 610031, China; 3 School of Animal Science and Technology, Guangxi University, Nanning 530004, China; 4 Center for Bio-Intelligent Manufacturing and Living Matter Bioprinting, Research Institute of Tsinghua University in Shenzhen, Tsinghua University, Shenzhen 518057, China; 5 Vernon Jansen Unit, Faculty of Medical and Health Sciences, The University of Auckland, Auckland 1023, New Zealand

**Keywords:** skin, wound, wound repair, extracellular vesicles, hydrogel, miRNA

## Abstract

Although extracellular vesicles (EVs) from mesenchymal stem cells have shown potential in skin wound repair, the diversity of EV sources and the optimization of delivery systems still need further exploration. This study is the first to demonstrate that extracellular vesicles from chemically induced mammary epithelial cells (CiMECs-EVs) possess distinct skin wound repair activity. To enhance the therapeutic efficacy of CiMECs-EVs and optimize their delivery efficiency, we innovatively combined them with a chitosan hydrogel to construct a composite repair system (CiMECs-EVs-chitosan hydrogel, CMECG). This system was then applied to a rat skin wound model. The results showed that CMECG significantly promoted the proliferation and migration of fibroblasts and mammary epithelial cells (MECs). In animal experiments, the relative wound closure efficiency of the control group was approximately 70% on day 14, while that of the CMECG group (loaded with 200 μg CiMECs-Exo) was enhanced to 90%, markedly accelerating the wound healing process. Histological analysis indicated that this system could effectively restore the structural continuity of various skin layers and significantly promote the synthesis and remodeling of collagen at the wound site. Mechanistically, the wound healing effect of CiMECs-EVs is closely associated with the endogenous miRNAs they encapsulate. These miRNAs can coordinately regulate cell proliferation, migration, and angiogenesis, modulate the inflammatory microenvironment, and inhibit excessive scar formation—thus regulating the entire repair process. This process involves multiple wound healing-related signaling pathways, including MAPK, PI3K-Akt, FoxO, TGF-β, and JAK-STAT. In summary, this study successfully constructed a novel EV-chitosan hydrogel repair system. This system is expected to provide an effective and innovative EV-based therapeutic strategy for the clinical treatment of skin wound repair.

## 1. Introduction

The skin serves as the body’s first protective barrier. Skin wounds often trigger infections, which may progress to chronic non-healing wounds [1]. Conventional methods for wound treatment have limited therapeutic efficacy. Meanwhile, cell-based therapies for skin tissue engineering carry risks of adverse reactions, such as immune rejection, infection, and allergies [2]. Extracellular vesicles (EVs)—tiny membranous vesicles that cells release—exhibit low immunogenicity and are free from ethical restrictions. These features make them a promising tool with impressive potential in the field of wound repair [1].

Wound repair encompasses three sequential yet overlapping phases: the inflammatory phase, the proliferative phase, and the tissue remodeling phase [3]. In recent years, accumulating evidence has demonstrated that EVs regulate all three phases to promote wound healing. During inflammation, EVs from mesenchymal stem cells (MSCs) drive M2 macrophage polarization to exert anti-inflammatory effects [4]. In the proliferative phase, Shu et al. reported that EVs secreted by human umbilical cord MSCs significantly enhance the proliferation of epidermal cells, including fibroblasts and epidermal keratinocytes [5]. Audigier et al. found that EVs from endothelial progenitor cells (EPCs) upregulate the expression of angiogenesis-related factors (e.g., VEGFA, FGF2, and Cox-2), thereby accelerating angiogenesis [6]. During tissue remodeling, Li et al. confirmed that EVs from human adipose-derived MSCs activate fibroblasts and increase collagen synthesis. These effects help prevent scarring and accelerate wound repair [7].

Despite these advances, the clinical translation of therapies based on extracellular vesicles (EVs) faces two critical bottlenecks. First, there is a lack of EV sources that have both high repair potency and accessibility. Second, delivery systems need optimization to preserve EV activity [8]. Mesenchymal stem cells (MSCs)—the most widely studied source of EVs—have issues including donor variability, limited capacity for in vitro expansion, and inconsistent therapeutic efficacy across batches [9]. Other sources (e.g., endothelial progenitor cells (EPCs) or keratinocytes) are either difficult to isolate or have narrow functional specificity [10]. These challenges highlight the urgency to identify novel, superior EV sources.

Hydrogels are hydrophilic network structures. They have good biocompatibility, degradability, and strong water-holding capacity, so they have broad application prospects in the medical field—particularly as carriers for EV delivery [11]. Loading EVs into hydrogels maintains their structural integrity and biological activity. At the same time, this loading enables efficient sustained release, which is key to achieving ideal efficacy during wound healing [12]. Additionally, hydrogels form a physical barrier. This barrier protects wounds from external bacterial infection [13]. Their hydrophilic network ensures uniform EV distribution, while their degradability avoids in vivo tissue damage [11]. Studies have shown that loading extracellular vesicles (EVs) derived from human umbilical cord mesenchymal stem cells (MSCs) into hydrogels can accelerate skin wound healing by inhibiting inflammatory responses and promoting collagen deposition [14]; Zhang et al. confirmed that the hydrogel-mediated sustained release of EVs from human umbilical cord MSCs accelerates wound healing in diabetic rat models [15].

Chitosan is a natural cationic polymer obtained by deacetylating chitin. It offers unique advantages for extracellular vesicle (EV) delivery. Chitosan has inherent functions, including hemostasis, antibacterial activity, promotion of wound healing, and inhibition of scar formation. It also has excellent biocompatibility, with no immunogenicity or stimulatory effects on the body [16]. Literature reports that mixing EVs from human gingival mesenchymal stem cells (MSCs) with chitosan promotes collagen deposition and neovasculature formation, which accelerates wound repair [17]. When chitosan is formulated into a temperature-sensitive hydrogel, it maintains a sol state at room temperature—this allows convenient administration. The gel time, temperature, and viscosity of the hydrogel can be adjusted by modifying component materials to different ratios. These adjustments help optimize adhesion and the kinetics of EV release [18].

Against this backdrop, our laboratory has innovatively established a technical system. This system is used for chemically inducing the transdifferentiation of somatic cells into mammary epithelial cells (CiMECs). We propose that EVs from CiMECs (CiMECs-EVs) represent a superior alternative to conventional EV sources for three key reasons: CiMECs-EVs exhibit epithelial lineage specificity. This specificity enables targeted regulation of re-epithelialization—a rate-limiting step in wound closure—via conserved epithelial cell communication pathways [19]. Mammary epithelial cells are highly accessible (e.g., from discarded lactation-related samples or minimally invasive biopsies). They can also be efficiently expanded and induced in vitro, which avoids the donor-dependent variability of MSCs [20]. Our preliminary data show that CiMECs-EVs are enriched with miRNAs. These miRNAs are implicated in cell proliferation and tissue regeneration, suggesting untapped repair potential.

Building on this, we hypothesize that combining EVs from CiMECs (CiMECs-EVs) with a temperature-sensitive chitosan hydrogel will synergistically enhance wound repair. The hydrogel will perform three key functions: first, it will protect CiMECs-EVs from degradation; second, it will enable sustained local release of CiMECs-EVs; third, it will maintain a moist healing microenvironment. Meanwhile, CiMECs-EVs—with their epithelial lineage specificity—will drive targeted regulation of repair processes. The specific objectives of this study are as follows: (1) to construct a composite system of CiMECs-EVs and chitosan hydrogel (CiMECs-EVs-chitosan hydrogel, CMECG); (2) to evaluate, in vitro, the effect of CMECG on the proliferation and migration of fibroblasts and mammary epithelial cells (MECs); (3) to validate, in vivo, the therapeutic efficacy of CMECG in two aspects: accelerating skin wound healing in rats and maintaining the integrity of skin tissue; (4) To preliminarily explore the regulatory mechanism of CMECG, with a focus on the miRNAs encapsulated in EVs. This work aims to achieve two goals: first, to confirm that CiMECs-EVs are a novel and effective source of EVs; second, to provide an optimized delivery strategy that combines EVs with hydrogel for clinical wound repair.

## 2. Results

### 2.1. Fibroblasts Are Transformed into CiMECs Through the Action of RepSox

**Figure 1:**
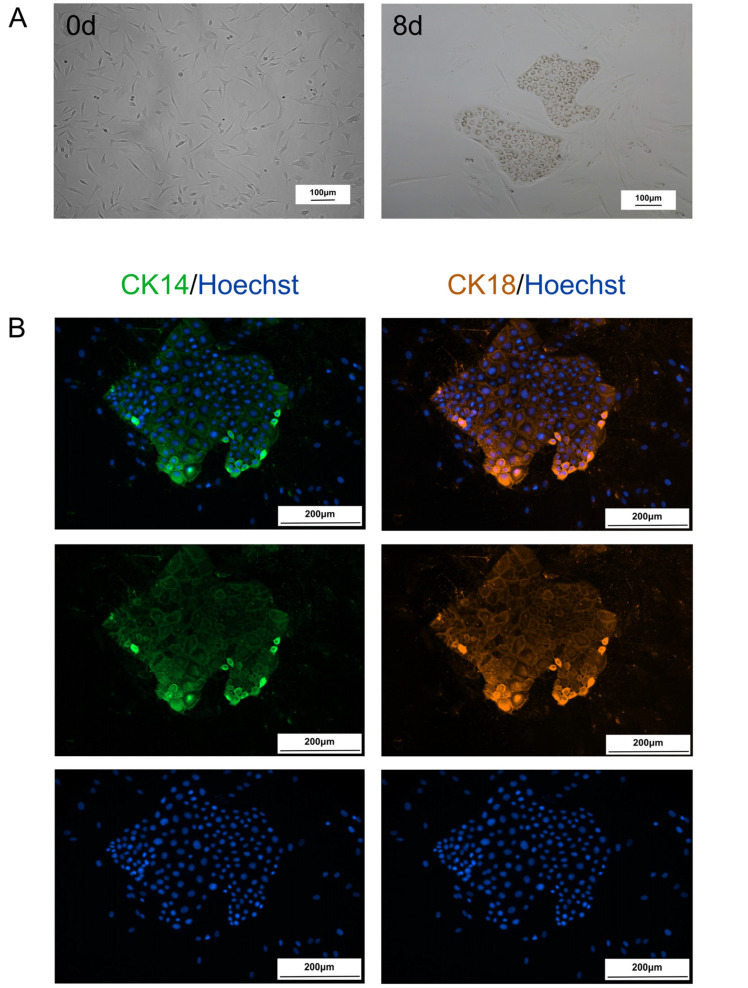
Fibroblasts were transformed into CiMECs via RepSox treatment: (**A**). Morphological changes in the cells during the induction process (day 0 to day 8). Scale bar, 100 μm. (**B**). Immunofluorescence staining of CiMEC-specific markers (CK14 and CK18). Scale bar, 200 μm.

We found that after fibroblasts were cultured in RepSox small molecule compound-induced medium for 8 days, the cells changed from a typical fibroblast-like long spindle shape to a colony-like shape of epithelial-like cell clusters, which presented a typical MEC morphology (Figure 1A) and expressed the related specific markers CK14 and CK18 of MECs (Figure 1B).

### 2.2. Extraction and Characterization of CiMECs-EVs

**Figure 2:**
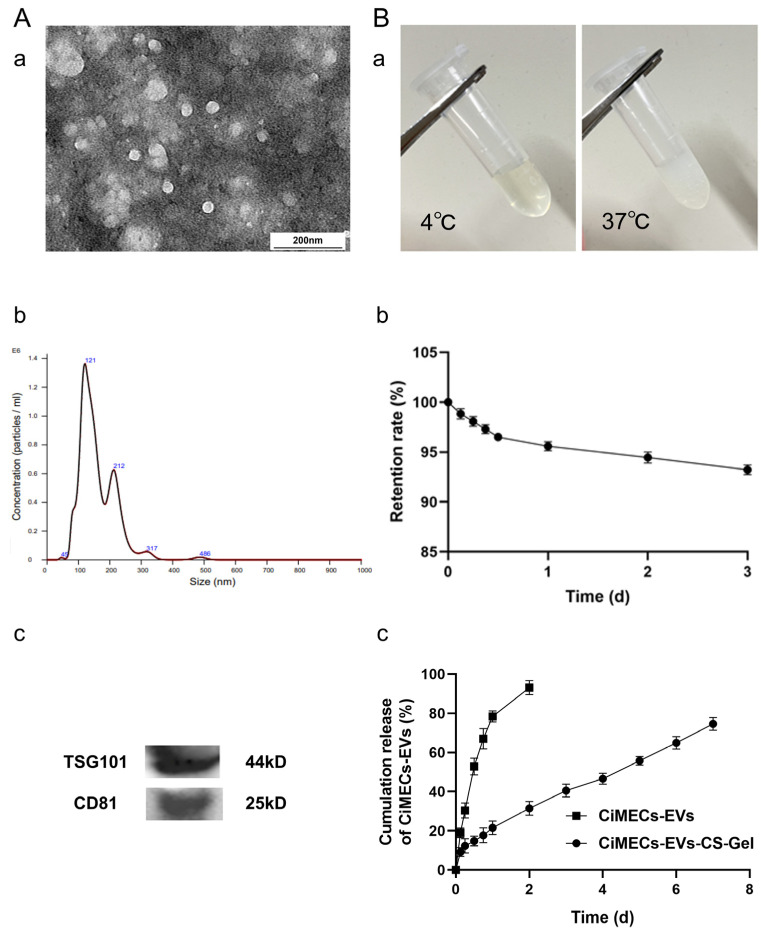
Identification of CiMECs-EVs and characterization of CMECG: (**A**). TEM, NTA, and WB identification of CiMECs-EVs. (**a**). TEM identification of CiMECs-EVs; (**b**). NTA identification of CiMECs-EVs; (**c**). WB identification of CiMECs-EVs. (**B**). Temperature sensitivity of CMECG and the retention and release effects of CiMECs-EVs: (**a**). state of CMECG at 4 °C and 37 °C; (**b**). retention effect of CMECG on CiMECs-EVs; (**c**). release effect of CMECG on CiMECs-EVs.

We used the differential centrifugation method to extract EVs from the collected CiMECs. Electron microscopy revealed that the extract presented a typical spherical structure (Figure 2A(a)). Nanoparticle tracking analysis revealed that the particle size of the extract was concentrated mainly at 121 nm (Figure 2A(b)). Western blot results revealed that the extract expressed the protein markers TSG101 and CD81 in the EVs (Figure 2A(c)). These results indicated that CiMECs-EVs were successfully isolated.

### 2.3. Preparation of the CMECG

We prepared CMECG via the ionic cross-linking method. The results showed that CMECG was liquid at 4 °C and transformed into a gel at 37 °C (Figure 2B(a)), indicating that it has good temperature sensitivity. The results of the retention experiments revealed that the retention rate of the EVs in the gel after 1 day was approximately 96%, the retention rate after 2 days was approximately 94%, and the retention rate after 3 days reached approximately 93% (Figure 2B(b)). The results of the release experiments revealed that, in the CiMECs-EVs group, the release of EVs was faster, with the cumulative release amount for 2 days accounting for approximately 95%, whereas the release of EVs in the CMECG group was slower, with the cumulative release amount for 2 days being approximately 30% and the cumulative release amount for 4 days. The release amount was approximately 45%, the cumulative release amount for 6 days was approximately 65%, and the cumulative release amount for 7 days was approximately 75% (Figure 2B(c)), indicating a slow release effect.

### 2.4. CMECG Can Promote the Proliferation of Fibroblasts/MECs

**Figure 3:**
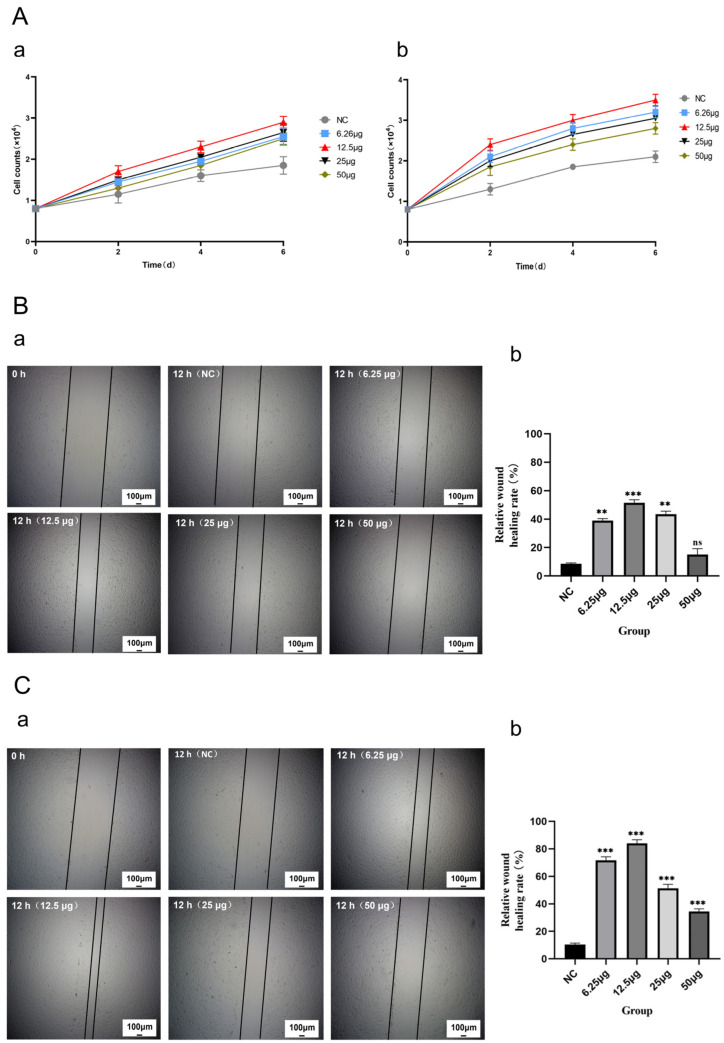
CMECG promoted the proliferation and migration of fibroblasts/MECs: (**A**). CMECG promoted the proliferation of fibroblasts/MECs: (**a**). effects of CMECG with different concentrations of CiMECs-EVs on the proliferation of fibroblasts; (**b**). effects of CMECG with different concentrations of CiMECs-EVs on the proliferation of MECs. (**B**). CMECG promoted the migration of fibroblasts: (**a**). Effects of CMECG containing different concentrations of CiMECs-EVs on the migration of fibroblasts; scale bar, 100 μm. (**b**). Quantitative statistical analysis of the scratch results. (**C**). CMECG promoted the migration of MECs: (**a**). Effects of CMECG containing different concentrations of CiMECs-EVs on the migration of MECs; scale bar, 100 μm. (**b**). Quantitative statistical analysis of the scratch results. Note: All experiments were performed with *n* = 3 independent experiments, each with 3 technical replicates. Statistical analysis was performed using One-way ANOVA followed by Tukey’s post hoc test. *** *p* < 0.001, ** *p* < 0.01. “ns” stands for “non-statistical significance”.

We evaluated the effect of CMECG on the proliferation of fibroblasts/MECs via the cell counting method and compared the effects of CMECG with different concentrations of CiMECs-EVs on the proliferation of fibroblasts/MECs. The results revealed that the proliferation rate of fibroblasts/MECs in the CMECG group with different contents of CiMECs-EVs was significantly faster than that in the hydrogel control group (NC group) over the same period, and the CMECG group containing 12.5 μg CiMECs-EVs had the fastest proliferation rate (Figure 3A(a,b)). In summary, the above experimental results showed that CMECG could significantly promote the proliferation of fibroblasts/MECs.

### 2.5. CMECG Can Promote the Migration of Fibroblasts/MECs

We evaluated the effect of CMECG on the migration of fibroblasts/MECs through cell scratch experiments and compared the effects of CMECG with different concentrations of CiMECs-EVs on the migration of fibroblasts/MECs. The effects of CMECG on the migration of both fibroblasts and MECs revealed that after 12 h, the scratch width of the CMECG group with different contents of CiMECs-EVs was narrower than that of the hydrogel control group (NC group) during the same period, and the CMECG group containing 12.5 μg CiMECs-EVs significantly narrowed the width of the scars (Figure 3B(a),C(a)). Image analysis of the scratch area revealed that the relative wound healing efficiency of the hydrogel control group (NC group) was approximately 10%, and the relative wound healing efficiency of the CMECG group containing 12.5 μg CiMECs-EVs against fibroblasts was approximately 50%, 5 times greater than that of the NC group. In contrast, the relative wound healing efficiency of MECs in the CMECG group containing 12.5 μg CiMECs-EVs was approximately 85%, which was 8.5 times greater than that of the NC group. The relative wound healing efficiency of the CMECG group with different contents of CiMECs-EVs was also significantly greater than that of the hydrogel control group (NC group) (Figure 3B(b),C(b)). In summary, the above experimental results showed that CMECG could significantly promote the migration of fibroblasts/MECs.

### 2.6. CMECG Can Accelerate the Repair of Wounded Skin in Rats

**Figure 4:**
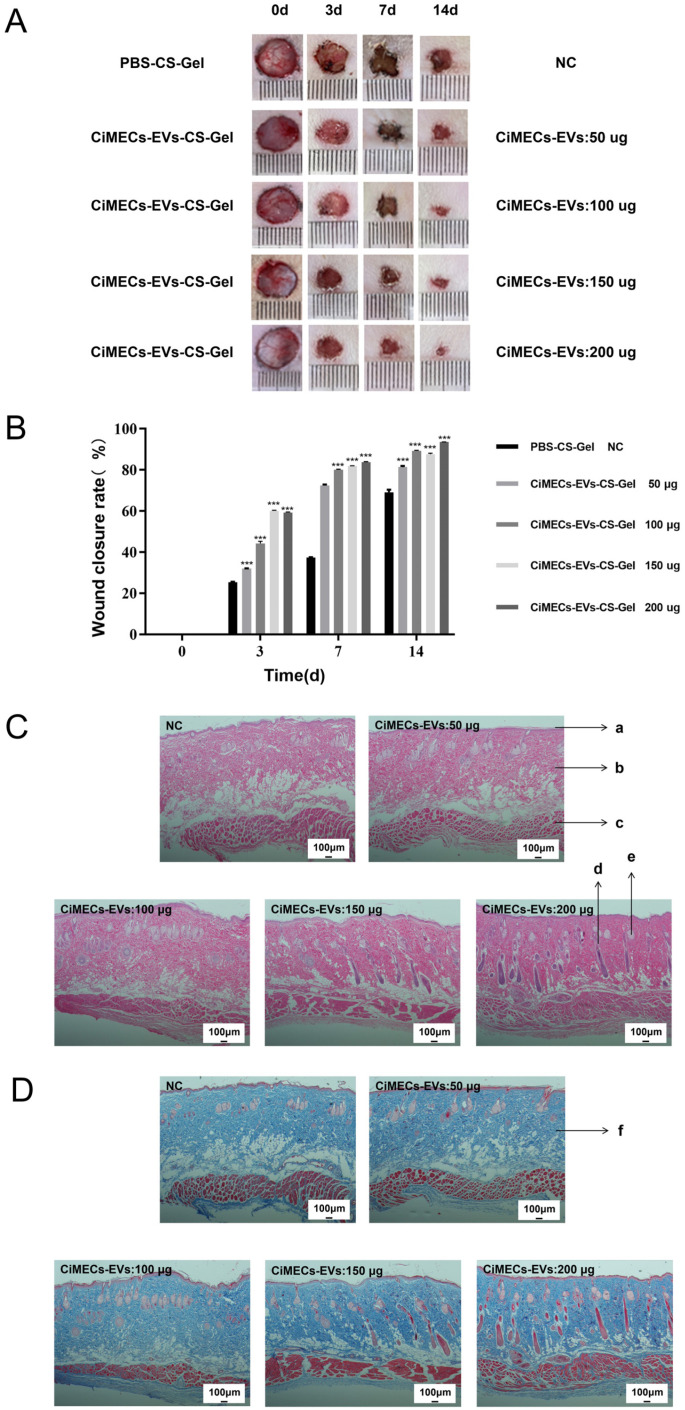
CMECG promoted the repair of skin wounds, the regeneration of skin tissues, and the formation of collagen in rats. (**A**). Wound closure status of rat wound skin repaired by CMECG with different concentrations of CiMECs-EVs. (**B**). Wound healing rate. (**C**). Histological analysis of wound callus tissue: HE staining. a: Epidermis b: dermis c: Dermatomuscular tissue d: Hair follicle e: Sebaceous gland; Scale bar, 100 μm. (**D**). Histological analysis of wound callus tissue: Masson staining. f: Collagen fibers (blue-stained portion). Scale bar, 100 μm. Note: All experiments were performed with *n* = 3 independent experiments, each with 3 technical replicates. Statistical analysis was performed using One-way ANOVA followed by Tukey’s post hoc test. *** *p* < 0.001.

We used a rat skin wound model to study the repair effect of CMECG on wounds. The results revealed that the wound area of the CMECG group with different contents of CiMECs-EVs was smaller than that of the hydrogel control group (NC group) over the same period, and for the CMECG containing 200 μg CiMECs-EVs, the wound area of the EV group was significantly smaller than that of the hydrogel control group (Figure 4A). Analysis of the wound area via ImageJ revealed that the relative wound healing efficiency of the NC group was approximately 70% on Day 14, and the relative wound healing efficiencies of the CMECG groups treated with 50 μg, 100 μg, 150 μg, or 200 μg of CiMECs-EVs were approximately 70%. There were significant differences at the 80%, 85%, 83%, and 90% levels (Figure 4B), indicating that CMECG significantly promoted skin wound repair.

We also evaluated the health of the wounds via HE and Masson staining. HE staining revealed that as the number of CiMECs-EVs increased, the number of restored hair follicles increased. Compared with those in the NC group, the hair follicles in the CMECG group contained more hair follicles, the sebaceous glands were more evenly distributed, and the structural continuity of each skin layer was greater, indicating that the skin wounds were effectively repaired. In the NC group, there were almost no hair follicles, the dermis was not completely restored, and the subcutaneous tissue was discontinuous (Figure 4C). In addition, the results of Masson staining revealed that, compared with that in the NC group, the expression of collagen in the CMECG group was more abundant, compact and orderly (Figure 4D). In general, among the samples in each group, those in the CMECG group containing 200 μg CiMECs-EVs presented more hair follicles, more sebaceous glands, greater structural continuity, and more abundant and tightly arranged collagen. These findings revealed that CMECG had the best reparative effect and confirmed that CMECG promoted skin tissue regeneration and collagen formation in skin wounds.

### 2.7. Mechanistic Study of CiMECs-EVs in Skin Wound Repair

To further explore the mechanism underlying the promotion of skin wound repair by CiMECs-EVs, we performed miRNA sequencing and bioinformatics analysis of CiMECs-EVs to reveal the relationship between EV-derived miRNAs and skin wound repair.

**Figure 5:**
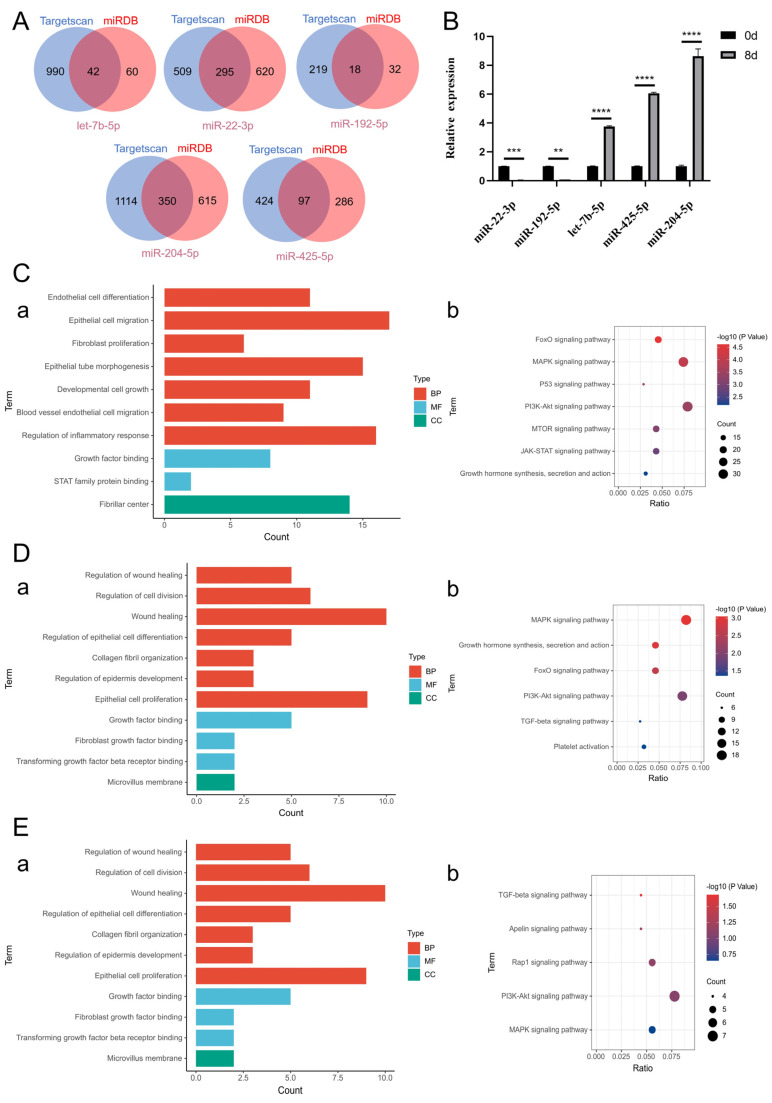
MiRNA sequencing and bioinformatics analysis revealed that the miRNAs present in CiMECs-EVs were closely related to skin wound repair. (**A**). Venn diagram of the predicted miRNA targets. (**B**). Validation results of differential miRNA quantitative PCR. Note: **** *p* < 0.0001, *** *p* < 0.001, ** *p* < 0.01. (**C**–**E**). GO functional analysis and KEGG pathway enrichment analysis of the target genes of let−7b−5p, miR−22−3p and miR−192−5p. (**a**). GO functional analysis of miRNA target genes; (**b**). KEGG pathway enrichment analysis of miRNA target genes.

**Figure 6:**
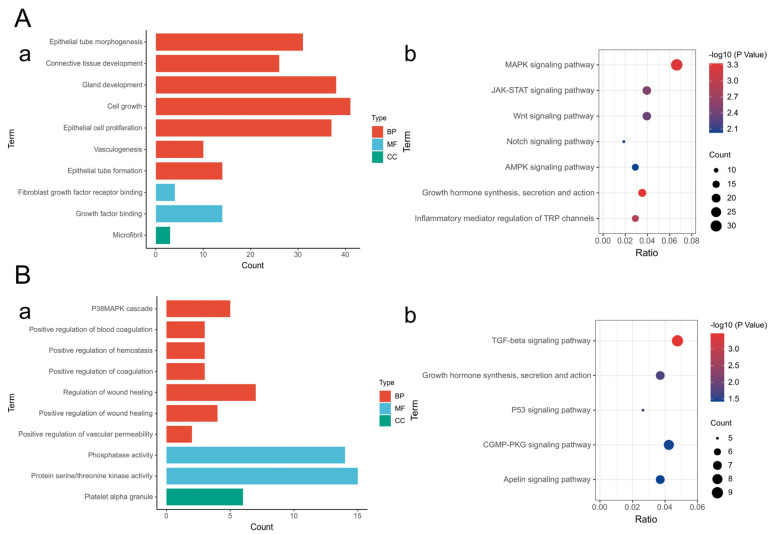
(**A**,**B**). GO functional analysis and KEGG pathway enrichment analysis of the target genes of miR−204−5p, and miR−425−5p. (**a**). GO functional analysis of miRNA target genes; (**b**). KEGG pathway enrichment analysis of miRNA target genes.

On the basis of the results of miRNA high-throughput sequencing and previously published literature, we selected 5 miRNAs (let-7b-5p/miR-22-3p/miR-192-5p/miR-204-5p/miR-425-5p) that presented significant changes in exosomes before and after induction and were related to skin wound repair for further investigation (Figure 5A). Compared with those before induction, the expression levels of let-7b-5p, miR-204-5p and miR-425-5p in exosomes after induction were upregulated, whereas the expression levels of miR-22-3p and miR-192-5p were downregulated (Figure 5B). The results of the quantitative analysis of differentially expressed miRNAs were consistent with the results of the differential analysis of the sequencing data, indicating that the sequencing data are authentic and valid and that the results of the analysis of differential miRNA expression before and after induction have certain reference values. In addition, the results of the GO functional enrichment analysis of the differential miRNA target genes revealed that the molecular functions of the miRNA target genes are related mainly to fibroblast growth factor binding, transforming growth factor receptor binding, platelet-derived growth factor binding, extracellular matrix structural components, and phosphatase activity; the involved biological processes include mainly the regulation of inflammation, fibroblast proliferation, epithelial cell proliferation, epithelial cell migration, angiogenesis, vascular endothelial cell migration, wound healing, and gland development; and the cellular components are associated mainly with the fiber center, fibrous collagen trimers, and platelet α granules (Figure 5C(a),D(a),E(a) and Figure 6A(a),B(a)). These biological processes are closely related to skin wound repair. The results of the KEGG pathway enrichment analysis revealed that the target genes of the differentially expressed miRNAs were significantly enriched in the MAPK signaling pathway, the PI3K-Akt signaling pathway, the FoxO signaling pathway, the TGF-β signaling pathway, and the JAK-STAT signaling pathway (Figure 5C(b),D(b),E(b) and Figure 6A(b),B(b)). These pathways have been confirmed to play important regulatory roles in skin wound repair.

## 3. Discussion

In our previous study, we innovatively established a technical system for inducing the transdifferentiation of somatic cells into chemically induced mammary epithelial cells (CiMECs) using the small-molecule compound Repsox, providing a new direction for autologous cell therapy of breast defects [21]. Building on this, the present study further isolated CiMEC-derived extracellular vesicles (CiMECs-EVs) from the culture supernatant of CiMECs. Verification via transmission electron microscopy (exhibiting a typical cup-shaped morphology), nanoparticle tracking analysis (showing a particle size distribution of 100–150 nm), and Western blotting (positive for CD81 and TSG101) confirmed that these vesicles conformed to the core characteristics of EVs [22]. To address the issues of easy diffusion and short half-life of free EVs in vivo, we prepared the CMECG (CiMECs-EVs-chitosan hydrogel) system using a reference ion cross-linking method [23]. Results showed that the local retention efficiency of EVs by this system was comparable to that reported in the literature [24], and its sustained release effect was significantly superior to other hydrogels [12], making it a stable delivery carrier for subsequent experiments.

Rapid wound healing relies on the proliferation, migration, and subsequent tissue regeneration of fibroblasts/mammary epithelial cells (MECs) [25]. Previous studies have confirmed that extracellular vesicles (EVs) derived from mesenchymal stem cells or fibroblasts can promote wound healing by regulating cellular activity [26], and the results of this study are consistent with these findings. Additionally, a unique dose–response relationship was observed: in in vitro experiments, the 12.5 μg CiMECs-EVs group exhibited the strongest pro-repair effect, while the effect weakened as the dose increased. It is hypothesized that high concentrations of EVs may lead to the saturation of cell surface receptors, thereby triggering negative signal feedback [27]. Furthermore, the optimal in vivo dose (200 μg) was higher than the optimal in vitro dose (12.5 μg). This is because inflammatory factors and proteases at the in vivo wound site can degrade EVs [28], the hydrogel degrades more rapidly, and the repair process involves multiple stages—all of which require a higher dose to meet therapeutic needs [29]. Overall, the pro-repair effect of CMECG was comparable to that of EV-hydrogel systems reported in the literature [30]. In the rat wound model, CMECG not only achieved an ideal wound closure rate but also promoted hair follicle regeneration and orderly collagen deposition, with effects similar to those of antioxidant-based wound repair [31].

miRNAs are core functional molecules in EVs and are widely involved in various stages of wound repair [32]. For example, let-7b can resolve inflammation by inducing the differentiation of macrophages into the anti-inflammatory M2 phenotype [33]; miR-204-5p can promote the proliferation of vascular endothelial cells while inhibiting apoptosis and inflammation [34]; miR-425-5p can enhance angiogenesis and skin re-epithelialization [35]; miR-192-5p can regulate collagen deposition and fibrotic hyperplasia in wounds [7]; and miR-22-3p can affect the formation of fibrotic scars by regulating myofibroblast differentiation [36]. The induction and generation of CiMECs is a process of epithelial formation, which theoretically is regulated by miRNAs that promote epithelial formation or proliferation. Through miRNA sequencing, this study found that CiMECs-EVs were also enriched with such functional miRNAs. Their target genes are involved in repair processes such as inflammation regulation, cell proliferation, and migration, and are significantly enriched in classical signaling pathways, including MAPK, PI3K-Akt, FoxO, TGF-β, and JAK-STAT—all of which have been confirmed to play important regulatory roles in skin wound repair [37]. These findings provide a theoretical basis for understanding the mechanism of wound repair. However, the study has limitations: first, there is a lack of functional verification for the identified miRNAs, and the mechanistic explanation remains at the level of correlative inference; second, the follow-up period was short (only 14 days post-operation), failing to evaluate long-term repair effects and biosafety, and thus cannot rule out potential issues such as scar contracture in the later stage.

The translational potential of CMECG is reflected in three aspects: first, CiMECs can be induced from autologous somatic cells, avoiding immune rejection, and the use of a single inducer enables simple operation and easy standardization [17]; second, the thermosensitive property of the hydrogel facilitates clinical local administration, and the release of EVs can be optimized by adjusting the degree of deacetylation [16]; third, it integrates local protection, sustained drug delivery, and targeted repair functions, which is in line with clinical treatment needs. Nevertheless, potential risks need to be addressed during translation: CiMECs are induced by small molecules, so their genomic stability needs to be verified; the current in vitro expansion efficiency of CiMECs is low, making it difficult to meet the clinical demand for EV quantity; and EVs may carry tissue-specific antigens, so the immune response after autologous/allogeneic infusion needs to be verified.

This study is the first to apply CiMECs-EVs to skin wound repair and confirmed that CMECG can accelerate wound healing by regulating cell activity and signaling pathways. Despite its limitations, this study provides a new EV source and delivery strategy for wound repair. Future research will focus on supplementing functional verification of key miRNAs, extending the in vivo follow-up period, and optimizing the large-scale culture process of CiMECs to promote its clinical translation, with the goal of providing an effective treatment for chronic refractory wounds.

## 4. Materials and Methods

**Cell culture**

Goat ear margin tissue blocks were collected from Guanzhong goats at the Guangxi Animal Husbandry and Veterinary Research Institute. Primary cells were isolated via the tissue block method. The cell culture medium used was DMEM (Gibco, Grand Island, NY, USA, Cat. #11965-092) + 10% fetal calf serum (WISENT, St-Bruno, QC, Canada, Cat. #086-150), the passaged cells were digested with trypsin (Gibco, Grand Island, NY, USA, Cat. #25200-072), and the digestion was terminated with 10% FBS in a total volume. After centrifugation, the cell suspension was added evenly to a new culture dish based on the basis of the sedimentation volume. The main instruments are shown in Table 1.

**Induction of CiMECs**

When the density of the attached fibroblasts reached approximately 40%, the medium was changed to RepSox induction medium (RepSox, Selleck Chemicals, Cat. No.: S7223, CAS No.: 446859-33-2; Source: Houston, TX, USA). Thereafter, the RepSox induction medium was changed every 2 days for 8 days. The RepSox concentration used was 10 μM. Induction medium recipes (100 mL as an example): Knockout DMEM/F12 (Life Technologies, Carlsbad, CA, USA, 11330-032): 40 mL; Neurobasal (Life Technologies, Carlsbad, CA, USA, 21103-049): 40 mL; KnockOut™ Serum Replacement (Gibco, Grand Island, NY, USA, 10828-028): 20 mL; 0.5% N2 (Invitrogen, Carlsbad, CA, USA, 17502048); 1% B27 (Invitrogen, Carlsbad, CA, USA, 17504044); Glutamine (100×) (Gibco, Grand Island, NY, USA, Cat. #25030-081): 1 mL; corresponding small molecule compounds were added according to the above concentrations. The main instruments are shown in Table 1.

**IF staining**

After fibroblasts were induced for 8 days, the cells were fixed in 4% paraformaldehyde for 20 min at room temperature, a blocking solution (PBS containing 100 mmol/L glycine and 0.3% BSA) was added, and then, 1% Triton X-100 was added and incubated at room temperature for 15 min. Then, a blocking solution (100 mmol/L glycine and 0.3% BSA in PBS buffer) was added for washing, and the samples were blocked with 1% BSA for 1.5 h at room temperature, followed by the addition of a primary antibody (Mouse monoclonal anti-CK14, NOVUS, Littleton, CO, USA, Cat#NBP2-45032, RRID:AB_3309227 and Mouse monoclonal anti-CK18, NOVUS, Littleton, CO, USA, Cat#NBP2-44951, RRID:AB_2811032), incubation at 4 °C in the refrigerator overnight, and then incubation at room temperature in the dark. After incubation with secondary antibody for 1.5 h (Alexa Fluor 488 Donkey Anti-mouse, Abcam, Cambridge, MA, USA, Cat#ab150109; RRID: AB_2571721 and Alexa Fluor 555 Donkey Anti-mouse, Abcam, Cambridge, MA, USA, Cat#ab150100; RRID: AB_2890133), images were taken under a fluorescence microscope and recorded. The main instruments are shown in Table 1.

**EV extraction**

The CiMECs culture medium collected on days 0, 2, 4, 6, and 8 during the induction process was placed in a sterile centrifuge tube. After thorough mixing, EVs were isolated and extracted using the differential centrifugation method. The specific procedure was as follows: centrifugation at 300× *g* for 10 min, 2000× *g* for 10 min, and 10,000× *g* for 30 min sequentially at 4 °C; subsequent filtration through a 0.22 μm filter; followed by centrifugation at 100,000× *g* for 70 min. The pellet was then resuspended in PBS and transferred to a sterile EP tube. The main instruments are shown in Table 1.

**Morphology of the EVs observed via transmission electron microscopy**

EVs were placed on copper grids for incubation at room temperature for 25 min, and then, 1% acetic acid staining solution was added dropwise and incubated in the dark for 2 min. Then, the staining solution was aspirated with filter paper. After the surfaces of the copper grids were dry, the samples were transferred to the sample stem for transmission electron microscopy. After the corresponding parameters are adjusted, pictures are taken and saved. The main instruments are shown in Table 1.

**Analysis of EV particle size via a nanoparticle tracking analyzer (NTA)**

The dynamic light scattering method is used to obtain the particle size distribution and quantity of the particles in the liquid suspension by emitting a laser beam with concentrated energy on the sample through a glass prism. The particle size range of the EVs was recorded and analyzed. The main instruments are shown in Table 1.

**Protein markers of EVs detected by Western blot**

Urea lysis buffer and 5× SDS loading buffer were added to the EVs, which were boiled for 5 min, after which the EVs and protein markers were added to the prepared loading wells of the gel for electrophoresis. After that, the gel was cut to an appropriate size, and the filter paper, PVDF membrane, gel, and filter paper were placed in the center of the membrane transfer instrument from top to bottom for membrane transfer. The PVDF membrane was subsequently incubated with 5% skim milk powder and blocked at room temperature for 2 h. The PVDF membrane was subsequently placed in a primary antibody mixture and incubated at 4 °C overnight (Mouse monoclonal anti-CD81, NOVUS, Littleton, CO, USA, Cat#NB100-65805, RRID: AB_962702 and Rabbit polyclonal anti-TSG101, Sigma-Aldrich, St. Louis, MO, USA, Cat#AV38773, RRID: AB_1858389). The next day, secondary antibody incubation was performed at room temperature for 2 h. Finally, the PVDF membrane was placed in the chromogenic agent, and the PVDF membrane was observed and photographed via an imager (ImageJ (Version 1.54i), NIH; downloaded from https://imagej.nih.gov/ij/download.html (accessed on 10 May 2024). The main instruments are shown in Table 1.

**Preparation of the CMECG**

Chitosan hydrogels were prepared via the ionic cross-linking method. A total of 0.5 g of chitosan powder (CS) was added to 20 mL of 0.1% acetic acid solution and stirred magnetically for 2 h. At the same time, 0.2 g of NaHCO_3_ powder and 1 g of β-glycerophosphate disodium pentahydrate (β-GP) were added to 2 mL of triple-distilled water for ultrasonic dissolution and then filtered through a 0.22 μm filter. Finally, 2 mL of the prepared β-GP solution containing NaHCO_3_ was added dropwise to the prepared 20 mL of CS solution. Then, the mixture was centrifuged at 2000 rpm for 10 s to obtain the chitosan hydrogel (CS-Gel). Subsequently, CiMECs-Exo were mixed with CS-Gel uniformly at a ratio of 1:4 to obtain CMECG.The main instruments are shown in Table 1.

**Retention effect of the CMECG**

The CiMECs-Exos were labeled according to the DiO fluorescence labeling instructions. Moreover, CMECG was prepared. Subsequently, 1.2 mL of PBS was added to the gel, which was placed on a constant-temperature shaker at 37 °C and 60 r/min. Samples (400 μL) were taken at 0, 3, 6, 9, 12, 24, 48, and 72 h, respectively, and fresh PBS was added to maintain a constant volume. The fluorescence values at 488 and 525 nm were measured on a microplate reader with a gain of 100, and the retention rate of CiMECs-Exo in CMECG was calculated. The main instruments are shown in Table 1.

**Release effect of CMECG**

CiMECs-EVs were labeled according to the instruction manual of DiO fluorescence labeling. The Transwell chamber was placed in a 24-well plate, the CMECG was prepared, and after it turned into a gel, it was placed in the upper chamber of the 24-well plate. At the same time, 1.2% PBS containing 10% fetal calf serum was added to the lower well of the 24-well plate. The 24-well plate was then placed in a thermostatic shaker at 37 °C and 60 r/min. A 400 μL sample was collected on days 0, 0.125, 3, 4, 5, 6, and 7, and new PBS was added at the same time. The fluorescence values at 488 and 525 nm were measured on a microplate reader to calculate the release rate of CiMECs-EVs in CMECG. The main instruments are shown in Table 1.

**The ability of CMECG to promote cell proliferation**

Fibroblasts/MECs were seeded into 96-well plates and counted via the cell counting method, and approximately 8 × 10^3^ cells were seeded in each well. After the fibroblasts/MECs had completely adhered, serum-free medium was added, and the wells were plated separately. CMECGs containing 6.25 μg, 12.5 μg, 25 μg, or 50 μg CiMECs-EVs were added for incubation. The dressing was changed once every two days. The hydrogel control group was used as the control group (NC group). The number of cells after d, 4 d, and 6 d was counted to evaluate the ability of CMECG to promote the proliferation of fibroblasts/MECs. Moreover, the proliferation-promoting effects of CMECs with different CiMECs-EV contents on fibroblasts/MECs were compared. The main instruments are shown in Table 1.

**CMECG promoted cell migration ability**

Fibroblasts/MECs were seeded into 96-well plates and counted via the cell counting method, and approximately 1 × 10^4^ cells were seeded into each well. When the cells were completely attached and the growth density reached more than 90%, a cell scratch experiment was performed to remove the cells. To evaluate the migration ability, CMECGs containing 6.25 μg, 12.5 μg, 25 μg, or 50 μg CiMECs-EVs were added to each well for incubation, and the dressing was changed once every two days. The hydrogel control group was used as the control group (NC group). After being cultured for 12 h, the 96-well plate was removed and observed under an inverted microscope, and the width of the scratch was recorded and photographed. ImageJ software (Version 1.54i) was used to quantitatively detect the migration distance of each group to evaluate the ability of CMECG to promote the migration of fibroblasts/MECs. Moreover, the ability of CMECs with different CiMECs-EV contents to promote the migration of fibroblasts/MECs was compared. The main instruments are shown in Table 1.

**Preparation of the rat skin wound model**

In this study, 5-week-old male SD rats weighing 150–200 g were used. After the rats were anesthetized via intraperitoneal injection of 3% pentobarbital sodium, their back hair was shaved with a razor, and a skin biopsy punch (diameter of 1 cm) was used to make a circular mark on the skin surface. Then, the complete skin was cut along the depression of the mark with surgical scissors. Using this method, two symmetrical skin wounds were made on the back of each rat at intervals of 1.5 cm. The main instruments are shown in Table 1.

**Assessment of wound healing ability**

For the skin trauma model, the rats were randomly divided into 4 groups, with 5 animals in each group. Full-thickness skin wounds were created on the left and right sides of the backs of the rats. The right wounds of each group were smeared with CMECGs with different contents of CiMECs-EVs, and the left wounds in each group were smeared with CMECGs with different contents of CiMECs-EVs. As a control, the nonEV group (NC group) was administered the same volume of PBS@CS-Gel once every 2 d. Pictures were taken on days 0, 3, 7, and 14, and images were taken via ImageJ software. The wound surface was measured. The main instruments are shown in Table 1.

**Histological evaluation**

The rats in each group were sacrificed after 14 days of CMECG. The wound tissues were excised with surgical scissors, fixed in 4% formaldehyde, dehydrated in ethanol, and embedded in paraffin, followed by histological sectioning and HE and Masson staining. After staining, the stained tissues were observed and photographed via a fluorescence microscope. The main instruments are shown in Table 1.

**HE staining**

The sections were placed in an oven at 60 °C for 3 h and then placed in xylene I and xylene II. After hematoxylin staining, the samples were rinsed with water, treated with a 1% hydrochloric acid alcohol solution for a few seconds, and then rinsed with purified water to turn them blue. The samples were stained with eosin staining solution. The sections were dehydrated sequentially in 75%, 85%, 95%, and 100% ethanol solutions; then, they were placed in xylene I and xylene II for 30 s each. After drying, the sections were sealed with neutral resin and then observed and photographed under a microscope. The main instruments are shown in Table 1.

**Masson staining**

The sections were dewaxed with water, stained with Weigert iron hematoxylin staining solution, washed thoroughly with Masson blue solution, washed with distilled water for 1 min, stained with Ponceau magenta staining solution, and washed in weak acid working solution (1%). After being washed with phosphomolybdic acid solution, the sections were washed with weak acid working solution and stained with aniline blue staining solution without washing. The sections were subsequently washed with weak acid working solution, dehydrated rapidly with 95% ethanol three times, cleared with xylene three times, and sealed with neutral resin. The sections were observed under a microscope and photographed. The main instruments are shown in Table 1.

**miRNA sequencing**

Total RNA was extracted from exosomes using RNA isolator Total RNA Extraction Reagent (Vazyme, Nanjing, Jiangsu, China, Cat#R401-01). The purity of the extracted RNA was determined using a Nanodrop One (with an A260/A280 ratio of 1.8–2.1), and its integrity was verified by an Agilent 2100 Bioanalyzer (with an RNA Integrity Number, RIN > 7.0). A total of 1 μg of qualified RNA was used for small RNA library construction with the Illumina TruSeq Small RNA Library Prep Kit (Illumina, San Diego, CA, USA, Cat. No. RS-200-0012), which involved the removal of large-molecular-weight RNA, ligation of 5′/3′ adapters, reverse transcription-polymerase chain reaction (RT-PCR) amplification, and purification of the 140–160 bp band. High-throughput sequencing was performed on the Illumina NovaSeq 6000 platform with single-end 50 bp reads, and each sample was sequenced to a depth of ≥20 million clean reads. Raw sequencing data were processed with Cutadapt to filter out adapters and abnormal fragments. Subsequently, the Bowtie software (Version 1.3.1) was used to align the processed data to the human GRCh38.p13 genome and miRBase 22.0 database to screen for sequences matching known miRNAs. Differentially expressed miRNAs between pre-induction and post-induction groups were identified using DESeq2 software (based on R 4.0.5) with the criteria of |log_2_ fold change| > 1 and adjusted *p*-value < 0.05. Three biological replicates were included in the experiment to ensure the reliability of the results. The main instruments are shown in Table 1.

**Differential miRNA target gene prediction**

The prediction of target genes for differentially expressed miRNAs in this study was mainly carried out via TargetScan and MIRDB (TargetScan, miRNA prediction, https://www.targetscan.org/vert_71/ (accessed on 10 March 2025) and MiRDB, miRNA prediction, https://mirdb.org/mirdb/index.html (accessed on 10 March 2025)).

**Target gene functional enrichment and pathway enrichment analysis**

An R script was used to perform GO functional enrichment analysis on the target genes, with a filtering criterion of a *p* value < 0.05 (R version 4.0.5, The R Foundation for Statistical Computing, https://www.r-project.org/ (accessed on 11 March 2025)). The DAVID database was used to perform KEGG pathway enrichment analysis on the target genes, with the filtering condition of a *p* value < 0.05.

**qPCR detection**

Through RNA extraction (RNA isolator Total RNA Extraction Reagent, Vazyme, Nanjing, Jiangsu, China, Cat#R401-01), cDNA was synthesized by reverse transcription. Using the cDNA as the template, along with primers and qPCR mixture, the expression levels of miRNAs before and after induction were detected by qPCR (miRNA 1st strand cDNA Synthesis Kit (by stem-loop), Vazyme, Nanjing, Jiangsu, China, Cat#MR101-01 and ChamQ SYBR qPCR Master Mix kit, Vazyme, Nanjing, Jiangsu, China, Cat. #Q711-02). The main instruments are shown in Table 1. The miRNA primers are shown in Table 2.

**Quantification and statistical analysis**

Statistical analysis of the quantified data was performed via GraphPad software (GraphPad Prism 9, GraphPad Software, https://www.graphpad.com/ (accessed on 12 March 2025)). Significance was calculated with Student’s *t* test or one-way ANOVA, unless otherwise stated. The data are presented as the means ± SEMs. * *p* < 0.05, ** *p* < 0.01, *** *p* < 0.001, **** *p* < 0.0001.

## Figures and Tables

**Table 1:** Main Instruments.

Main Instruments	Brand	Model	Origin
CO_2_ Incubator	Thermo	371	Waltham, MA, USA
Ultra-clean Workbench	Suzhou Jiade Purification	JB-CJ-1000FX	Suzhou, Jiangsu, China
Inverted Microscope	Nikon	Eclipse MA100N	Tokyo, Japan
Centrifuge	Eppendorf	5702/R/RH	Hamburg, Germany
Constant Temperature Water Bath	Stuart	SWB1	Stone, Staffordshire, UK
Gel Imaging System	Tianneng	1600	Shanghai, China
Inverted Fluorescence Microimaging System	Nikon	Eclipse Ti2	Tokyo, Japan
Cell Imaging Multimode Microplate Reader	Bio Tek	Bio Tek Synergy Neo2	Winooski, VT, USA
Refrigerator	Haier	DW-86L286	Qingdao, Shandong, China
Ice Maker	Haier	HZB-12A	Qingdao, Shandong, China
Pipette	Eppendorf	Research plus	Hamburg, Germany
Drying Oven	Lichen	DZF-6020AB	Shanghai, China
Autoclave	Panasonic	TOMY SX-500	Osaka, Japan
Purification System	Millipore	Milli-Q integral	Burlington, MA, USA
Refrigerated Centrifuge	Eppendorf	5910 Ri	Hamburg, Germany
Ultracentrifuge	BeckMan	Optima XE-100	Brea, CA, USA
Transmission Electron Microscope	Hitachi	HT7800	Tokyo, Japan
Magnetic Stirrer	Lichen	78-1	Shanghai, China
Vortex Mixer	Lichen	LC-Mixer-RD	Shanghai, China
Electron Microscope	FEI	Tecnai G2 20	Hillsboro, OR, USA
Nanoparticle Tracking Analyzer	ZetaView	ZetaView PMX 110	Berlin, Germany
Electric Constant Temperature Shaker	IKA	THZ-103B	Staufen, Germany
Microplate Reader	Biotek	Synergy H1	Winooski, VT, USA

**Table 2:** miRNA primers.

The miRNA Primers		
Primer: *let-7b-5p* RT: GTCGTATCCAGTCAGGGTCCGAGGTATCGCACTGGATACGACAACCAC	This paper	N/A
Primer: *let-7b-5p* Forward: GCGCGTGAGGTAGTAGGTTGT	This paper	N/A
Primer: *let-7b-5p* Reverse: AGTGCAGGGTCCGAGGTATT	This paper	N/A
Primer: *miR-22-3p* RT: GTCGTATCAGTGCAGGGTCCGAGGTATCGCACTGGATACGACACGTT	This paper	N/A
Primer: *miR-22-3p* Forward: GCGAAGCTGCCAGTTGAAG	This paper	N/A
Primer: *miR-22-3p* Reverse: AGTGCAGGGTCCGAGGTATT	This paper	N/A
Primer: *miR-192-5p* RT: GTCGTATCAGTGCAGGGTCCGAGGTATTCGCACTGGATACGACGGCTGT	This paper	N/A
Primer: *miR-192-5p* Forward: GCGCGCTGACCTATGAATTG	This paper	N/A
Primer: *miR-192-5p* Reverse: AGTGCAGGGTCCGAGGTATT	This paper	N/A
Primer: *miR-204-5p* RT: GTCGTATCAGTGCAGGGTCCGAGGTATCGCACTGGATACGACAGGCAT	This paper	N/A
Primer: *miR-204-5p* Forward: CGCGTTCCTTTTGTCATCCT	This paper	N/A
Primer: *miR-204-5p* Reverse: AGTGCAGGGTCCGAGGTATT	This paper	N/A
Primer: *miR-425-5p* RT: GTCGTATCAGTGCAGGGTCCGAGGTATTCGCACTGGATACGACTCAACG	This paper	N/A
Primer: *miR-425-5p* Forward: GCGAATGACACGATCACTCC	This paper	N/A
Primer: *miR-425-5p* Reverse: AGTGCAGGGTCCGAGGTATT	This paper	N/A
Primer: *U6* Forward: TCTGCTTTACTGCCGACCAG	This paper	N/A
Primer: *U6* Reverse: CAGGCTGATGTGGAAGGAGG	This paper	N/A

## Data Availability

The sequencing data (miRNA-seq) required to reproduce these findings are available for download from the GEO (https://www.ncbi.nlm.nih.gov/geo (accessed on 12 March 2025); Access ID: GSE206772).
